# Methodological challenges of analysing COVID-19 data during the pandemic

**DOI:** 10.1186/s12874-020-00972-6

**Published:** 2020-04-14

**Authors:** Martin Wolkewitz, Livia Puljak

**Affiliations:** 1 Institute of Medical Biometry and Statistics, Faculty of Medicine and Medical Center, University of Freiburg, 79104 Freiburg, Germany; 2 Center for Evidence-Based Medicine and Health Care, Catholic University of Croatia, Ilica 242, 10000 Zagreb, Croatia

## Editorial

On March 11, 2020, the World Health Organization (WHO) declared that COVID-19 can be characterized as a pandemic [1]. The disease is caused by the novel coronavirus SARS-CoV-2, which rapidly overwhelmed the entire world. The virus was first described in China in December 2019, in early January it was already characterized, and already on January 30, 2020, the outbreak was declared a Public Health Emergency of International Concern, which later evolved into a pandemic [1].

Devastating and unpredictable spread of COVID-19 throughout the world has caused unprecedented global lockdowns and immense burden for healthcare systems. The WHO called for immediate research actions including “immediately assess available data to learn what standard of care approaches are the most effective” and “evaluate as fast as possible the effect of adjunctive and supportive therapies” [1].

This pandemic is now an enormous challenge for researchers, clinicians, health-care workers, epidemiologists and decision-makers. BMC Medical Research Methodology would like to contribute to this global endeavour by setting up a collection of articles called “Methodologies for COVID-19 research and data analysis”. As Guest Editors of the Collection, we would like to offer our views regarding methodological challenges where researchers can help.

## Statistical challenges of analysing COVID-19 data

Statistical models will play a major role in “fighting panic with information” [2] to avoid or at least minimize the risk of bias which is a common threat in clinical and epidemiological studies. In this article, we describe the most striking challenges for statisticians and data analysts who want to provide support in this pandemic with their expertise.

### Getting proper clinical data of active and closed COVID-19 cases

After the outbreak in Wuhan, China (available as open access epidemiological data [3]), clinical data can be prospectively collected in a cohort study design. Merging and cleaning of data from large multi-centre hospitals is crucial and requires sophisticated data management. Artificial intelligence and deep learning algorithm might be suitable to tackle this challenge. Data security, patients consent, ethics statements are essential in non-pandemic situation but they are bureaucratic barriers to get rapid access to clinical data. Pandemic situations require specific handling of these issues and should be discussed on national level.

We have to distinguish between active (still hospitalized) and closed (discharged or dead) COVID-19 cases. Case report forms (CRF) for patients with suspected or confirmed COVID-19 are needed to collect and store their data in a standardised way. There are two main initiatives which created protocols for the investigators, the ‘International Severe Acute Respiratory and emerging Infection Consortium (ISARIC)’ (isaric.tghn.org) and the ‘Lean European Open Survey on SARS-CoV-2 Infected Patients (LEOSS)’ (leoss.net). In these two initiatives, it is planned that only closed COVID-19 cases are stored.

### Understanding the complexity of clinical endpoints

Endpoints in patients with severe pneumonia are challenging [4]. For COVID-19 patients, the most relevant clinical endpoints are the admission to intensive care, invasive ventilation and survival. Less relevant endpoints include the need of supportive oxygen. The analysis of these endpoints requires complex models which handles the time-dependent dynamic of the data.

### Understanding common statistical pitfalls in clinical epidemiology

Clinical data are highly time-dependent and require advanced statistical methods to avoid common pitfalls such as selection, length, immortal-time and competing risk bias [5–8].

### Developing appropriate analysis strategies

In the same way as data should be collected in a standardised way, data should also be analysed in a standardised way. Statisticians are encouraged to develop suitable analytical strategies to analyse data which were collected from standardised protocols (such as ISARIC and LEOSS).

### Communicating statistical effects and distinguishing them from artefacts

Communicating statistics, especially in hectic times during a pandemic, is very challenging. Statisticians are encouraged to support this with clear and transparent statements.

### Learning from similar studies about SARS, MERS and influenza A(H1N1pdm09)

As in other outbreaks such as SARS in 2002–2003, clinicians are confronted with new diseases for which there is limited knowledge of effective treatment options [9]. Since there is no targeted agent for COVID-19 in such an early outbreak phase, repurposing of available anti-viral drugs and corticosteroids is discussed [9–16], based on case series [17–23]. Until promising targeted randomized controlled trials exist, it is expected that large observational clinical studies will be performed to evaluate potential treatment effects as it was done, for instance, for SARS, MERS and influenza A(H1N1pdm09) on hospital mortality [24–27]. Observational studies cannot replace randomized controlled trials due to their limited ability to draw causal conclusions. However, they can be used to stimulate further research on the effectiveness of potential treatment options.

### Updating reporting guidelines for observational studies during a pandemic

In pandemic situation, rapid and valid information flow and reporting is crucial. Long-lasting reporting guidelines might do more harm than good. Specific reporting guidelines are needed for pandemic settings.

### Statistical support for randomized trial

The first randomized trial about Lopinavir–Ritonavir for Covid-19 patients has already been published and showed no promising effect [28]. Statistical expertise is needed to understand potential effects on the complexity of clinical endpoints.

## Other methodological challenges in research on COVID-19

Beyond challenges related to data analysis, there are many other methodological challenges related to research on SARS-CoV-2 and COVID-19.

### Searching for relevant information sources

We are witnessing tremendous growth of articles published on this topic, already counting in thousands. For methodologists and researchers in the field of evidence synthesis, the challenge will be searching for the relevant information sources. Creating specialized, publicly accessible collection of studies with original studies about COVID-19 can surely help in this. For example, WHO has set up a collection of articles about COVID-19, compiled in a publicly available database. On March 30, 2020 this database had already included 3294 articles.

Source of those articles is described by WHO as [quote]: “*We update the database daily from searches of bibliographic databases, hand searches of the table of contents of relevant journals, and the addition of other relevant scientific articles that come to our attention*” [29]. However, by 6 April 2020 it was not publicly reported which databases and journals are searched for this purpose. The WHO web site offers several crude search filters available, for searching these articles. The WHO also offers filtering for “Newest updates”, but it is not clear how new are the newest updates, i.e. there is no search by date. The articles in the database can be downloaded, but cursory look at those articles indicates that the majority of them do not have original data; instead it appears that the majority are news, commentaries and opinions. Thus, it would be useful to separate articles in this database that actually report original data. At the time when this article went to publication, multiple other collections of evidence on COVID-19 were being announced and set up, indicating that multiple teams globally are creating the same or similar evidence collections, leading to needless waste of human resources.

### Synthesizing evidence rapidly

In a world where each day brings hundreds of new articles on a hot topic, conducting evidence synthesis will be particularly challenging. Systematic reviews are considered by many as the highest-level of evidence in the hierarchy of evidence in medicine, but their production often takes years [30, 31]. However, multiple systematic reviews about COVID-19 have already been published. It remains to be seen what is the quality of those rapidly produced systematic reviews.

Producing evidence syntheses on a short time scale usually requires cutting corners with methodology, and for this reason, rapid reviews have evolved. Rapid reviews are conducted with a condensed timeline, sacrificing certain aspects of systematic review methodology for speed [32]. Pilot study has shown, for example, that rapid research needs appraisal can be conducted within 5 days in the case of an infectious disease outbreak [33]. However, it has also been shown that transparency and inadequate reporting are the major limitations of rapid reviews [34].

### Ensuring adequate quality of published research

Journal editors are currently under pressure to publish relevant articles on COVID-19 quickly, which has been described as “rather maddening”. It has been argued that this could also be advantageous in a long run, as it can help journals to become more efficient in future.

However, haste is likely to be detrimental to the quality of publications. Speed is not necessarily a friend of good science. Articles may be assembled too quickly, publishing processes may be hastened, and quality of peer-review may not be adequate. Anecdotal reports indicate that highly specialized experts in the field may be swamped with requests for peer-review that they are unable to accommodate, which may lead to inviting less specialized peer-reviewers, to the detriment of manuscript quality check. We will need to wait to find out how many corrections and retractions there will be for journals published hastily on the topic of COVID-19, and whether methodological and reporting quality of those articles will be lower compared to the articles on other topics. In the times of emergency, researchers should still pay attention to transparency and adequate reporting of their research, to ensure its reproducibility.

### Data sharing

To enable analysis of data gathered during COVID-19 pandemic, principles of open science and raw data sharing will be of utmost importance. Global norms have been proposed [35] for data sharing during global health emergencies, and it remains to be seen whether researchers will be more likely to share their raw data publicly in articles covering COVID-19.

In conclusion, there are many methodological challenges related to producing, gathering, analysing, reporting and publishing data in condensed timelines required during a pandemic. We certainly did not mention all of them, but we hope that researchers willing to contribute to research methodology related to COVID-19 will help us address those other issues as well. It is customarily said that each crisis is also an opportunity, and therefore we hope that the BMC Medical Research Methodology will have an opportunity to publish research articles that will help the humanity win the battle against SARS-CoV-2.

## Data Availability

Not applicable.
